# Probiotics in Extraintestinal Diseases: Current Trends and New Directions

**DOI:** 10.3390/nu11040788

**Published:** 2019-04-05

**Authors:** Despoina E. Kiousi, Athanasios Karapetsas, Kyriaki Karolidou, Mihalis I. Panayiotidis, Aglaia Pappa, Alex Galanis

**Affiliations:** 1 Department of Molecular Biology and Genetics, Faculty of Health Sciences, Democritus University of Thrace, Alexandroupolis 68100, Greece; despkiou1@mbg.duth.gr (D.E.K.); a.karapetsas@dundee.ac.uk (A.K.); kyriakikarolidou@gmail.com (K.K.); apappa@mbg.duth.gr (A.P.); 2 Department of Applied Sciences, Northumbria University, Newcastle Upon Tyne NE1 8ST, UK; m.panagiotidis@northumbria.ac.uk

**Keywords:** probiotics, gastrointestinal, skin, bone, neuronal disease, microbiota

## Abstract

Probiotics are defined as live microorganisms that when administered in adequate amounts confer a health benefit to the host. Their positive supplementation outcomes on several gastrointestinal disorders are well defined. Nevertheless, their actions are not limited to the gut, but may also impart their beneficial effects at distant sites and organs. In this regard, in this review article we: (i) comprehensively describe the main mechanisms of action of probiotics at distant sites, including bones, skin, and brain; (ii) critically present their therapeutic potential against bone, skin, and neuronal diseases (e.g., osteoporosis, non-healing wounds and autoimmune skin illnesses, mood, behavior, memory, and cognitive impairments); (iii) address the current gaps in the preclinical and clinical research; and (iv) indicate new research directions and suggest future investigations.

## 1. Introduction

Probiotics are defined as live microorganisms with health promoting properties. Although, the term “probiotics” was introduced by Lilly and Stillwell in 1965, Elie Metchnikoff, the Russian immunologist and Nobel Laureate in Medicine, was the first to describe their beneficial effects more than a century ago. Metchnikoff linked regular consumption of yogurt to the delay of the aging process and the promotion of longevity. His theory, presented in his book “The Prolongation of Life”, published in 1907, stated that putrefactive bacteria present in the gut release toxins and other harmful substances to the host. Administration of host-friendly bacteria found in yogurt could counteract this action, restore intestinal balance, and enhance human health. Nowadays, FAO and WHO (Food and Agriculture Organization of the United Nations and the World Health Organization) define probiotics as “live microorganisms that, when administered in adequate amounts, confer a health benefit on the host” [1].

A plethora of studies have demonstrated the positive outcomes of probiotic supplementation on several gastrointestinal disorders, such as antibiotics-associated diarrhea, irritable bowel syndrome, necrotizing enterocolitis, ulcerative colitis, lactose intolerance, and colorectal cancer [2]. Several articles reviewed their mechanisms of action, which include modulation of the immune system, induction of anti-inflammatory and anti-oxidant responses, competitive pathogen exclusion, as well as production of anti-microbial substances [3,4]. Additionally, probiotics could exert anti-proliferative effects in colon cancer cells by inducing apoptosis and cell-cycle arrest [5,6].

There is growing evidence that human microbiota can exert effects at sites quite distant from the niches in which they reside. For instance, it was shown that gut bacteria can affect memory and anxiety of animal models [7]. Interestingly, low microbiota diversity in the gut has been associated with increased asthma at school age [8]. In this regard, probiotic action is not limited to the gut, but also imparts their beneficial effects at distant sites and organs. Thushara et al., reviewed experimental and clinical studies of probiotic action against cardiovascular disorders, and an update of their effects on metabolic diseases was provided by Aggarwal et al. [9,10]. Moreover, it has been demonstrated that probiotic consumption results in production of anti-inflammatory agents that promote bone health and integrity and protect against primary (estrogen-deficiency) and secondary osteoporosis [11]. Similarly, probiotics have also been tested for their wound-healing abilities, protection against UV-induced photodamage, and also for alleviation of symptoms in skin diseases, such as dermatitis and psoriasis [12]. One of the most exciting research areas is their potential functional impact on the nervous system and neurological diseases. Studies in animal models have shown that consumption of probiotics can lead to decreased rate of stress- and anxiety-related symptoms [13]. Furthermore, probiotics have been documented in preclinical studies to suppress pro-inflammatory and oxidative damage responses in the brain, and thus, minimize the symptoms of neurodegenerative and demyelinating diseases [14]. However, as clinical evidence that supports probiotic action at distant sites is limited, no specific recommendations and medical guidelines for appropriate administration are available [15]. In this vein, the aims of this review are: (i) to present the main mechanisms of action of probiotics at distant sites, including bones, skin, and brain; (ii) to comprehensively describe and evaluate their therapeutic potential against bone, skin, and neuronal disorders (e.g., osteoporosis, non-healing wounds and autoimmune skin illnesses, mood, behavior, memory, and cognitive impairments); (iii) to address the current gaps in the preclinical and clinical research; and (iv) to indicate new research directions and suggest future investigations.

## 2. Probiotics and Bone Diseases

Bone formation is a complex process that requires the integration of the immune, hormonal, and neuronal systems. Hormone-associated bone damage, such as primary osteoporosis, as well as autoimmune diseases of the bones, including rheumatoid arthritis, are directly linked to improperly regulated inflammatory responses. Indeed, inflammation is detrimental to bone integrity, as it induces the production of matrix and cartilage degrading enzymes from chondrocytes and synoviocytes. Evidently, approaches targeting inflammation could be promising in managing bone disorders. In this context, probiotics with anti-inflammatory potential may induce the production of immunosuppressant cytokines, which activate the regulatory T-lymphocyte (Treg) population and reduce the formation of osteoclasts, resulting in the protection of bone integrity [16].

### 2.1. Sex-steroid-Associated Bone Loss—Primary Osteoporosis

Sex-steroid-associated bone loss is caused by hormonal and immunological imbalances. Primary osteoporosis represents the most common sex-steroid-associated bone disease. It is characterized by estrogen deficiency, which impairs cellular calcium uptake, weakens the gut barrier, and consequently causes increased permeability, which leads to endotoxemia and overactivation of immune responses. As a result, high levels of pro-inflammatory and osteoclastogenic cytokines are released in the gut lumen and the bone marrow, causing trabecular bone loss [17]. Probiotics counteract the detrimental effects of the hormonal deficit in various ways, as shown by in vitro and in vivo studies. *Lactobacillus helveticus* (LBK-16H) milk fermented products, isoleucyl-prolyl-proline (IPP) and valyl-prolyl-proline (VPP), were shown to promote osteoblastic bone formation and intracellular calcium accumulation in mouse osteoblast cells [18]. In addition, *L. helveticus* fermented milk prevented bone loss by decreasing bone turnover and increasing the bone mineral density in ovariectomized (OVX) rats [19]. Probiotic supplementation in ovariectomized female mice reversed cortical bone loss by decreasing CD4+T cell counts and the release of anti-inflammatory factors, such as tumor necrosis factor-α (TNF-α) and interleukin-1β (IL-1β), whilst increasing the production of osteoprotegerin, an important regulator of osteoclast development and function [20]. Anti-osteoclastogenic effects were also induced in rats fed with a diet rich in lipids after the consumption of *B. longum* fermented broccoli [21]. Moreover, the probiotic strain *L. reuteri* ATCC 6475 suppressed the expression of pro-inflammatory and pro-osteoclastogenic cytokines locally and in the bone marrow in ovariectomized mice [22]. Similarly, *L. rhamnosus* GG (LGG) decreased local and systemic inflammation and affected positively gut barrier function, thus protecting female sex-steroid deficient C57BL6/J mice against bone loss [23]. 

Other studies examined the morphological and biomechanical changes in bones of animal models after probiotic consumption. The animal bones were harvested post-mortem and examined using plasma emission spectrophotometry, fracture tests, and X-ray microcomputed tomography. In this context, *L. paracasei* subsp. *paracasei* NTU 101 and *L. plantarum* NTU 102 fermented soy milk increased bone density and thickness in female BALB/c ovariectomized mice. Of note, fermentation increased milk levels of calcium, vitamin D_3_, and isoflavone levels [24]. Similarly, *B. longum* alone, or in combination with yacon flour, a food with prebiotic potential, increased bone mineral content and strength in male Wistar rats compared to control rats. In addition, the combination of the two interventions resulted in elevated concentration of the propionic acid, while *B. longum* alone increased butyrate levels in the cecum [25]. It is well-established that short chain fatty acids (SCFAs), such as propionic acid and butyrate, maintain the integrity of the gut epithelium function by upregulating transcription of tight junction proteins and by limiting mucosal inflammatory responses [26,27]. Concomitantly, SCFAs promote bone health by inhibiting the differentiation of osteoclasts and decelerating bone resorption, as shown by in vitro and in vivo experiments [28].

Preliminary results from clinical studies have confirmed the beneficial effects of probiotics on bone diseases (Table 1). In a recent clinical trial, fifty osteopenic postmenopausal women over the age of 50 consumed GeriLact, a mixture of seven probiotic strains, for six months. Probiotic consumption influenced total hip and blood markers of bone turnover and resorption, including serum bone-specific alkaline phosphatase, osteocalcin, collagen type 1 cross-linked C-telopeptide, parathyroid hormone, vitamin D, and the proinflammatory cytokines, TNF-α and IL-1β. The overall positive result of GeriLact consumption was the deceleration of bone turnover rate [29]. In another study, healthy menopausal women who consumed tablets containing the probiotic stain *Bacillus subtilis* C-3102 exhibited increased bone mineral density accompanied with distinctive microbiological shifts in fecal samples. In particular, the *Fuseobacterium* population was significantly decreased, while species from the genus *Bifidobacterium* were found in greater concentrations. These changes in gut populations were attributed to reduced pro-inflammatory responses and inhibited osteoclast activity, ultimately favoring bone integrity and promoting the preventative potential of *B. subtilis* C-3102 on bone loss [30]. Nonetheless, further studies are required to validate the beneficial effects of this strain on osteoporotic patients. Finally, osteoporotic males that consumed kefir, a probiotic-enriched fermented dairy product, had increased femoral neck bone mineral density [31] (Table 1).

### 2.2. Autoimmune Diseases of the Bone

Rheumatoid arthritis (RA) is a multifactorial chronic autoimmune inflammatory disease. Its onset is often linked with exposure to various enteropathogens. Post-infection, antigenic cross-reactivity and the hyperproduction of inflammatory cytokines induce autoimmunity that leads to osteoclastic bone resorption and low bone mineral density. In addition, RA and inflammatory bowel disease (IBD) are oftentimes co-morbid. IBD-induced inflammation loosens the gut barrier and results in mass presentation of antigens to the intestinal immune system. It has been reported that probiotic supplementation could be effective in preventing and alleviating autoimmune bone disease. In particular, mice with collagen-induced arthritis (CIA) are widely used as experimental models of RA. CIA mice that were fed or inoculated with *L. helveticus* SBT2171 had dampened immune responses, ultimately resulting in alleviation of arthritis. This was mainly due to the decrease in auto-reactive B and T lymphocytes in draining lymph nodes, serum type II collagen (CII)-specific antibodies, and inflammatory cytokines [36]. Similarly, mice that were fed with a mixture of probiotics, zinc, and coenzyme Q10 exhibited alleviated arthritis symptoms that were attributed to the downregulation of proinflammatory cytokines [37]. However, a few clinical studies have yielded the expected results so far (Table 1). In a recent study, the use of probiotics in patients with knee osteoarthritis was investigated. In this group of patients, daily consumption of skimmed milk containing *L. casei* Shirota demonstrated lower scores in the Western Ontario and McMaster Universities Osteoarthritis Index and Visual Analog Scale tests, while serum high-sensitivity C-reactive protein (hs-CRP), an indicator of musculoskeletal inflammation, was decreased [32]. Interestingly, the researchers had previously established that *L. casei* Shirota accelerated healing of distal radius fractures [33]. Additionally, Zamani et al. showed that administration of a probiotic mixture consisting of *L. acidophilus, L. casei*, and *B. bifidum* normalized the levels of inflammatory markers and eased the clinical manifestations of RA [34]. In the same manner, women suffering from RA that consumed *L. casei* 01 daily for two weeks experienced improved drug responsiveness to disease-modifying anti-rheumatic drugs [35]. Nevertheless, further preclinical and clinical studies are required to delineate the health promoting effects of probiotics and the underlying molecular mechanisms related to specific bone diseases. 

## 3. Probiotics and Skin Health 

The interplay of hormonal, neuronal, and inflammatory signaling has a great impact on skin health. Psychological distress alters skin physiology by stimulating pro-inflammatory responses [38]. Indeed, acne, a common skin condition among adolescents and young adults, is correlated with neurogenic skin inflammation, which alters the functionality and survival of mast cells and induces the production of vasodilatory and proinflammatory factors. In addition, psychological stress upregulates secretion of prolactin, which in turn determines keratinocyte proliferation and sebum production by sebaceous glands [39]. Similarly, the onset of autoimmune skin diseases, such as psoriasis, and of allergic disorders, such as atopic dermatitis, is correlated to chronic inflammation and mast cell degranulation. The underlying, prolonged inflammatory responses induce keratinocyte apoptosis, contributing to the distinctive skin manifestations of these disorders. Current therapeutic approaches are either strenuous for the patient or have little effect. Probiotic bacteria with anti-inflammatory properties have the potential to be of therapeutic benefit for people suffering from neurogenic skin inflammation or autoimmune skin diseases. Nevertheless, more clinical evidence is needed to support their routine use in medical practice. Likewise, probiotics that protect the keratinocytes from oxidative stress or that induce skin re-epithelization could be of invaluable importance towards non-healing wounds. In addition, it has been shown that specific probiotic strains prevent skin aging by reversing photodamage. This finding highlights the potential of probiotics as supporting agents in skin health.

### 3.1. Neurogenic Skin Inflammation, Acne Rosacea, and Acne Vulgaris

Neurogenic skin inflammation arises from stress-induced over-expression of inflammatory cytokines and affects hair and skin by inhibiting hair growth and keratinocyte proliferation, respectively. It has been demonstrated that *L. reuteri* BM36301 supplementation on female C57BL/6 mice resulted in active hair growth, counteracted the hair follicle regression, and halted the recruitment of activated macrophages and dendritic cells perifollicularly [40]. In another study, investigators using an ex vivo skin explant model and showed that cell-free extract (CFE) from *B. longum* exhibited anti-inflammatory properties and protected skin from physical and chemical aggression [41].

Changes in skin microbiota composition and simultaneous bacterial overgrowth in the small intestine are quite common among individuals with acne rosacea. Indeed, *Propiobacterium acnes* overpopulation has been recorded in acne patients. Antibiotics targeting *P. acnes* are conventionally used to resolve acne. Alternatively, it has been reported that enhancement of *Staphylococcus epidermidis* skin population excludes *P. acnes* from sebaceous hair follicles. The antimicrobial effects of *S. epidermidis* are attributed to the production of SCFAs that exert direct microbicidal actions against *P. acnes* [42]. Interestingly, stimulation of *S. epidermidis* growth can be achieved by strain-specific *Lactobacillus* supplementation. Indeed, it has been reported that *L. brevis* DSM17250 secrets a peptide that stimulates proliferation of *S. epidermidis*. As a result, *L. brevis* DSM17250 supplementation may have indirect antimicrobial effects on skin pathogens [43]. Other probiotic strains have been reported to inhibit *P. acnes* directly. Results from in vitro experiments showed that strains *L. casei* NCFB 161, *L. acidophilus* NCFB 1748, *L. plantarum* DSM 12028, *L. gasseri* NCFB 2233, and *Lactococcus lactis* NCIMB 6681 exerted antimicrobial effects against *P. acnes*, which were enhanced when combined with prebiotics [44]. Similarly, *Lactococcus* sp. HY 449 produced a bacteriocin that was effective in limiting the pathogen in vitro and when applied topically in human patch tests [45]. 

Probiotic supplementation could also be employed for the alleviation of inflammation, a key aspect of acne onset. In one study, the probiotic strain *Escherichia coli* Nissle 1917 was administered orally to patients with acne and other intestinal-borne dermatoses. After the intervention period, the probiotic group had normal immunoglobin-A (IgA) counts, decreased proinflammatory cytokines levels, and normalized gut function. In addition, the skin lesions were significantly smaller or fully recovered [46]. Insulin signaling is a determining factor in post-adolescence acne vulgaris onset. Insulin, insulin-like growth factor-1 (IGF-1), and defective nuclear transcription factor forkhead box protein O1 (FOXO1) promote acne by stimulating sebaceous lipogenesis and androgen signaling. According to a recent clinical study, consumption of *L. rhamnosus* SP1 for 12-weeks led to decreased IGF-1 and increased FOXO1 gene expression that ultimately resulted in alleviation of acne manifestations in adult patients [47].

### 3.2. Psoriasis and Atopic Dermatitis

Chronic inflammation is a crucial factor for the development of autoimmune diseases. Specifically, pathological T-cells that reside on the skin of psoriatic patients overproduce IL-17 in response to IL-23, triggering the production of pro-inflammatory mediators IL-1β, IL-6, IL-8, TNF-α, and chemoattractants from keratinocytes. These signaling molecules sustain chronic skin inflammation and cause epidermal hyperplasia, the main trait of psoriatic plaques [48]. A novel therapeutic approach to tackle skin auto-immune diseases could be the utilization of immunomodulatory probiotic strains. In this vein, a BALB/c mice psoriasis model was used to evaluate the therapeutic potential of *L. pentosus* GMNL-77. Following application of this probiotic strain on skin lesions, pro-inflammatory cytokines were downregulated, the spleen size and differentiation of T-cells were decreased, and erythema and inflammation signs were alleviated [49]. In the same context, psoriasis patients that consumed *B. infantis* 35624 exerted a decrease in the proinflammatory markers IL-6, TNF-α, and serum CRP. These effects could be attributed to the probiotic-induced proliferation of Tregs. However, these findings were not directly linked to distinct clinical improvement [50]. Finally, in a clinical study investigating the beneficial effects for skin lesions of probiotics in patients with inflammatory bowel disease, a reduction of psoriasis skin lesions occurrence was observed in patients that consumed VSL#3, a probiotic preparation of eight live freeze-dried bacterial species [51]. 

Atopic dermatitis (AD) is the result of a Th1/Th2 leukocyte population imbalance that leads to excessive mast cell degranulation and a Th2-mediated allergic response. Phenotypically, this translates to skin erythema, hemorrhage, and itching that could be triggered by both genetic and environmental factors. Most of the studies on the alleviation of AD focus on two parameters; inflammation and the composition of gut and skin microbiota. Several studies on AD have employed probiotic strains that exhibit anti-inflammatory properties. Kim et al., administered a probiotic mixture of *L. casei, L. plantarum, L. rhamnosus, B. lactis*, and sodium butyrate to BABLB/c mice *per os*, following their sensitization with whey protein [52]. It was demonstrated that probiotic supplementation switched T cell differentiation towards Th1 and Treg populations and concomitantly, the microbiota composition was altered favoring the reduction of type I hypersensitivity [52]. Similarly, *L. plantarum* IS-10506 attenuated the levels of specific inflammation markers, such as IL-4, IL-17, and interferon-γ (IFN-γ), and increased the expression of immunomodulatory factors Forkhead box P3 (Foxp3+) and IL-10 in BALB/c mice, as well as in pediatric AD patients that had orally received this probiotic strain. These changes were accompanied by reduction in the Scoring Atopic Dermatitis Index (SCORAD) [53]. A significant decrease in Th2-mediated inflammatory responses and SCORAD scores were also induced by oral *L. acidophilus* L-92 administration on adult AD patients [54]. In the same context, the consumption of *L. salvarius* LA307 and *L. rhamnosus* LA305 inhibited skin inflammation in hairless SKH-1 mice and constituted a decrease in hyperplasia, immune cell skin infiltration, and hyperkeratosis, suggesting their potential benefits for AD patients [55]. *Lactococcus chungagensis* CAU 28T, a probiotic derived from activated sludge, displayed anti-inflammatory, anti-allergic, and anti-bacterial properties that lowered the molecular markers of AD and attenuated its clinical manifestations on NC/Nga mice, an inbred AD murine model, that were fed with this strain [56]. Similarly, oral administration of tyndallized and freeze-dried *L. rhamnosus* ICDD 3201 on Nc/Nga mice lowered IgE counts, resulting in the neutralization of AD symptoms [57]. 

AD skin lesions are frequently colonized by high loads of *S. aureus*. Therapeutic interventions limiting this pathogenic population result in clinical improvement of skin manifestations. It has been shown that *L. johnsonii* NCC 533 promoted the expression of antimicrobial peptides and inhibited adhesion of *S. aureus* to a reconstructed human epidermis (RHE) model in vitro [58]. In this text, *S. aureus*-positive AD patients who participated in an open-label multicenter study experienced improvement of lesion skin appearance after topical application of a lotion containing heat-treated *L. johnsonii* NCC 533 cells. Such a positive outcome was linked to the reduction of *S. aureus* population [59].

### 3.3. Chronic Conditions and Non-Healing Wounds

Non-healing wounds are a threat to diabetics, the elderly, the obese, and patients with chronic burn wounds. When applied topically, probiotics either selectively exclude pathogenic microorganisms (that deteriorate the wounds) or induce re-epithelization and collagen formation. In this light, researchers engineered a patch containing nitric oxide gas-producing *L. fermentum* 7230 and tested it on infected and ischemic rabbit wounds, assessing its wound-healing potential. Indeed, increased collagen synthesis and blood flow to the wounds was recorded [60]. Probiotic strains that reportedly induce keratinocyte migration and proliferation are LGG and *L. reuteri*, [61]. In addition, it has been shown that LGG enhanced tight-junction barrier function of human primary keratinocytes by inducing the increase of tight-junction protein levels of claudin 1, occludin, and ZO-1 [62]. Probiotic strains with antimicrobial properties contribute positively to the management of chronic wounds. In this context, probiotics that adhere to keratin exhibited antimicrobial properties against *E. coli*, *P. acnes*, and *Pseudomonas aeruginosa*. Furthermore, the same strains inhibited early biofilm formation without affecting mature biofilms in vitro [63]. In addition, *S. aureus* colonization could be combated by strain-specific application of LGG [64], as well as from skin commensals *S. epidermidis* [65] and *S. hominis* [66]. 

### 3.4. Aging Skin

Aging skin is characterized by increased pH, oxidative stress, and matrix metalloprotease activity that translate in wrinkle formation, dehydration, and discoloration. Accumulating preclinical and clinical evidence suggests that probiotics could potentially counteract phenotypic skin alterations, restore stratum corneum flexibility, and enhance hair quality after local or oral administration [67]. Environmental factors, such as Ultra-Violet (UV) irradiation, accelerate skin aging through skin dehydration and wrinkle formation. Probiotics appear to have regenerative effects on UV-induced photodamaged skin. Oral administration of *L. acidophilus* reduced wrinkle formation caused by UVB irradiation in a mouse model [68], while *Bifidobacteria breve* B-3 supplementation restored skin hydration and reversed tight junction and basal membrane photodamage [69]. In this context, Bouilly-Gauthier et al., investigated the ability of a supplement containing *L. johnsonii* and carotenoids to reverse early UV-induced photodamage in healthy women. The intervention was beneficial, as it restored the responses of the skin immune system [70]. Alternatively, other interventions were aimed toward the activation of the endogenous, protective mechanisms. For example, *Vitreoscilla fuliformis* altered the expression of a mitochondrial oxide dismutase, thus reducing UV-induced oxidative stress in vitro [71]. Another study reported that fermented acanthopanax koreanum root extract from *L. plantarum* and *B. bifidum* had anti-senescence and anti-oxidant effects on human skin fibroblast cells, greater than the non-fermented extract. The treated fibroblasts exhibited reduced expression of p53 and p21Cip1/WAF1 cell cycle proteins, and of matrix metalloproteinases, MMP-1, and MMP-3. These effects were mediated by the probiotic-induced activation of the mitogen-activated protein kinase (MAPK) signaling pathway [72]. Lastly, probiotics can be used for skin rehabilitation after cosmetic procedures. Fractional CO_2_ laser resurfacing treatments are used to reduce the signs of aging, but have an unpleasant healing process that includes inflammation, edema, erythema, and crusting. Post-operatively, the patients need to apply, topically, broad-spectrum antibiotics, sunscreen, and hyaluronic acid masks, though none of these alleviate inflammation [73]. Zoccali et al., employed CERABEST™, a cream that contains probiotic-derived compounds and recorded a statistically significant reduction of postoperative skin inflammation and swelling after topical application on the treated areas. However, probiotics have minimal compatibility with other components of skin creams. Moreover, they can be used only for a few days after production, as there is a rapid decline of their organoleptic properties. This is the main reason that to date, there is not any functional skin care product available in the market for commercial use [74]. A summary of the clinical studies investigating the beneficial effects of probiotics on skin disorders is presented in Table 2.

## 4. Probiotics and the Nervous System

Mood and behavior disorders may be caused by a chemical imbalance in the brain and are directly linked to plasma corticosterone levels and deregulation of the hypothalamus-pituitary-adrenal (HPA) axis. Neuroinflammation and autoimmunity provoke the onset of Alzheimer’s disease (AD), Multiple Sclerosis (MS), and Parkinson’s disease (PD) by disrupting the blood-brain barrier or inducing neuronal death. Conventional anti-depressants and anxiolytics have considerable side effects, while current therapeutic interventions against neurodegenerative diseases have limited therapeutic efficacy. Investigation and assessment of the potential of probiotics in preventing the symptoms or alleviating comorbidities against these diseases have been the goals of several clinical studies (Table 3).

### 4.1. Mood and Behavior Disorders

Stress- and depression-related behaviors are triggered by hormonal or neurotransmitter imbalances, such as high blood corticosterone and reduced brain serotonin levels, respectively. The effects of probiotics on these disorders are evaluated in preclinical animal models of chronic stress and depression, such as maternally separated or restrained mice and rats. Maternal separation is a strong early life stressor that disrupts the hypothalamus-pituitary-adrenal (HPA) axis, deregulating major physiological processes, such as stress response, gut function, and immunity. Two commercially available *Lactobacillus* strains, *L. rhamnosus* R0011 and *L. helveticus* R0052, were tested for their potential to restore the activity of HPA axis in maternally separated rat pups. After a 20-day dose regimen, plasma corticosterone (an indicator of the HPA axis function) was decreased to basal levels. Subsequently, the alteration of corticosterone concentration resulted in the alleviation of stress-related behaviors [88]. Likewise, supplementation of C57B1/6 male breast-fed, maternally separated mice with *Bifidobacterium pseudocatenulatum* CECT 7765 reduced blood corticosterone levels. In addition, this early-life probiotic intervention lowered stress-induced intestinal inflammation and gut hyper-catecholaminergic activity, exhibiting long- and short-term outcomes in brain biochemistry throughout the adult life of mice [89]. Chronic restraint models are employed to study depression and chronic stress. To this end, depressive and anxiety behavior was investigated in Sprague-Dawley (SD) rats after 21-day restraint regimens that were either exposed to *L. helveticus* NS8 or citalopram (an antidepressant drug). The rats that consumed the probiotic strain exhibited decreased incidence of chronic depression compared to those that consumed citalopram, as evidenced by behavioral assays. These findings were attributed to the (i) probiotics-mediated regulation of hormonal responses, (ii) increase of the anti-inflammatory interleukin, IL-10, and (iii) restoration of catecholamines and brain-derived neurotrophic factor (BDNF) levels in the hippocampus [90]. Anxiety alleviation, learning, and memory enhancement of anxious-born adult male BALB/c mice after probiotic supplementation with *B. longum* 1714 and *B. breve* 1205 was evaluated in a series of behavioral tests [91,92]. It was shown that the two strains resulted in different outcomes. Specifically, *B. longum* 1714 induced anti-depressant like behaviors together with better learning and memory, while *B. breve* 1205 alleviated anxiety behaviors. Nonetheless, no significant changes in corticosterone levels were observed after either intervention [91,92]. In a clinical study, major depressive disorder patients consumed a probiotic capsule containing freeze-dried *L. acidophilus*, *L. casei*, and *B. bifidum* for 8 weeks. At the end of the intervention, the probiotics group showed reduced inflammation and oxidative stress and experienced positive clinical outcomes [75].

Anxiety triggered by imbalances in serotonergic activity results in obsessive-compulsive disorder (OCD) phenotype. BALB/c mice that were pretreated with LGG before induction of the OCD phenotype did not exhibit OCD-like behaviors in behavioral testing. The beneficial outcomes of this intervention could be attributed to anxiety alleviation rather than to restoration of the neurological mechanisms implicated in OCD-onset. In this context, LGG could be administered alone or in combination with fluoxetine, an anti-depressant used for human OCD symptom alleviation [93]. Chronic fatigue syndrome (CFS) is a multifactorial disease with a wide spectrum of manifestations, many of which correlate to gut microbiota composition and function. In a clinical study, CFS patients experienced alleviation of anxiety symptoms after the consumption of *L. casei* strain Shirota (LcS). In addition, the participants’ microbiota was enriched with *Lactobacillus* and *Bifidobacteria* species post-treatment [76].

Studies have shown that probiotic consumption could protect predisposed individuals from chronic anxiety and depression. In this line, the efficacy of *L. helveticus* R0052 and *B. longum* R0175 was tested on healthy human volunteers. The volunteers’ clinical mental state was evaluated by global scores of hospital anxiety and depression scale (HADs), and the global severity index of the Hopkins symptoms checklist (HSCL-90). A positive score of mental health was recorded that was linked to changes in the hormonal state. Particularly, urinary free cortisol was reduced, indicating a visible effect on the host’s physiology. In addition, these strains exhibited anti-inflammatory properties, which might reflect the normalization of hormonal responses [77]. A clinical study showed that *B. longum* 1714 consumption improved cognitive performance, alleviated stress, and enhanced visuospatial memory and frontal midline mobility of healthy human adults by changing electrical brain activity, as recorded by electroencephalography (EEG) [78]. Likewise, the consumption of a mixture of probiotics (*B. animalis, Streptococcus thermophiles, L. bulgaricus,* and *L. lactis*) changed the brain electrical pattern in healthy female volunteers, as evaluated by functional magnetic resonance imaging (fMRI). The probiotics group exhibited decreased activity in the somatosensory cortex, the insula, and the periaqueductal faces after viewing emotionally evocative faces or scenes. This translates to a different emotional response compared to the placebo group [94]. Furthermore, probiotics with anxiolytic properties could be consumed by healthy individuals throughout stressful periods with beneficial effects. In this regard, in a clinical study, a number of students were recruited before their school exams to evaluate the anxiolytic potential of LcS. The participants consumed fermented milk containing LcS or placebo for 8 weeks before exams. LcS alleviated stress-induced changes in leukocyte gene expression and abdominal dysfunction. The mental state of the subjects was evaluated on the visual analogue state (VAS), and by measuring salivary cortisol 1 day before examination, which peaked only in the placebo group. Apart from alleviating visceral pain and abdominal symptoms, LcS suppressed the proliferation of the *Bacteroidaceae* family, which is oftentimes observed in IBS patients, and increased α-diversity, a marker of microbial community diversity that is linearly correlated to the balance of the gut microecosystem [79]. Other studies have demonstrated that probiotic strains limit anxiety manifestations, such as gut dysfunction [80], flu-like symptoms [81], and insomnia [82], as well as post-partum depression and anxiety [83] (see Table 3 for more details). Lastly, the possible effects of probiotic supplementation on the mental state of professional athletes were described in a recent systematic review. Exercise-induced stress behaviors are triggered by the psychological pressure of competitive athleticism and are possibly side-effects of a diet based on low consumption of plant-polysaccharides (that athletes are usually subjected to). This diet is associated with reduced gut microbiota diversity and functionality, causing deregulation of the HPA axis. It was suggested that a balanced diet containing pro- and prebiotics is capable of restoring microbiota structure and function, resulting in the resolution of stress disorders in athletes [95]. 

### 4.2. Visceral Nociception

Visceral pain refers to a wide range of pain sensations in the abdominal area and it is most prevalent among women. Nociceptors are scattered across the organs and communicate pain sensations to the brain by activating certain neuronal circuits. The vagus nerve is widely accepted as the main highway of signal transduction. In a recent study, Kaelberer et al., showed a direct connection between enteroendocrine cells and vagal nodose neurons [96]. Nociceptors can be triggered by a variety of stimuli, both endogenous and exogenous. Most of these stimuli, such as immune responses, microbiota configuration, and parasympathetic activity may be manipulated by probiotics. In a preclinical study, visceral hypersensitive Wistar-Kyoto (WKY) and visceral normosensitive SD rats were fed with *L. salivarius* UCC118, *B. infantis* 35624, or *B. breve* UCC2003 to evaluate their pain responses after colorectal distention. The rats exhibited increased pain threshold and decreased pain behaviors following *B. infantis* 35624 administration. The changes in pain perception were corticosterone-independent and did not affect rat behavior [97]. Rousseaux et al., showed that probiotic treatment of colon adenocarcinoma cell lines, HT29, changed the expression of analgesic opioid and cannabinoid receptors. Specifically, *L. acidophilus* NCFM and *L. salivarius* Ls-33 increased the transcription of the opioid receptor gene, whereas only *L. acidophulus* NCFM managed to upregulate expression of cannabinoid receptors [98]. To confirm the results in vivo, *L. acidophulus* NCFM cells were administered to BALB/c and SD rats. Immunohistochemical analysis revealed that the probiotic strain induced the expression of the analgesic receptors in gut epithelium. Conclusively, rats receiving probiotic supplementation exhibited increased pain thresholds during colorectal distention [98]. 

### 4.3. Neurodegenerative and Demyelinating Diseases 

Alzheimer’s disease, Multiple Sclerosis, and Parkinson’s disease share a common immunological basis. Chronic activation of the neuroimmune system, immunosenescense, and high levels of circulating proinflammatory cytokines result in neuronal death and disruption of the blood-brain barrier continuity, contributing to the manifestations of the diseases. Probiotics with anti-inflammatory and anti-oxidant potential have been studied for their beneficial effects against neurodegenerative and demyelinating disease onset, manifestations, and comorbidities. 

Probiotics may decelerate AD onset and minimize its manifestations by suppressing brain pro-inflammatory and oxidative responses, while improving cognition and memory. In this context, AD male Wistar rats that received *L. acidophilus, L. fermentum, B. lactis,* and *B. longum* in tap water exhibited decreased amyloid plaque size, and improved spatial memory, orientation, and mood. In addition, malondialdehyde and superoxide dismutase reached optimal levels in the hippocampi, contributing to the clearance of plaques. Interestingly, control rats did not exhibit any improvement in memory and cognition, indicating that the use of the probiotic strains did not enhance neuronal function in healthy animals [99]. Similarly, administration of SLAB51 probiotic formulation to transgenic AD mice preserved brain physiological function by activating Sirtuin-1 (SIRT1)-related pathways [100]. SIRT1 is a NAD+-dependent protein deacetylase with established neuroprotective potential, as it reduces ROS levels in the brain by activating superoxide dismutase 2 and catalase [101]. Probiotics targeting neuroinflammation are also effective in alleviating AD manifestations. Oral administration of *B. breve* A1 to AD mice prevented cognitive dysfunction and ameliorated neuronal inflammation through SCFA production [102]. Interestingly, SCFAs stimulate glutamate signaling, resulting in the elevation of brain γ-aminobutirc acid (GABA) levels [103]. Likewise, CFT-Swiss healthy male mice supplemented with *Enterococcus faecium* CFR 3003 and LGG had increased cytosolic GABA and dopamine (DA) levels [104]. More specifically, mice that consumed *E. faecium* CFR 3003 exhibited elevated DA levels in the cortex, while mice supplemented with LGG showed higher concentrations of DA in the striatum. *E. faecium* CFR 3003 induced the production of GABA in every brain region investigated (cortex, hippocampus, and striatum), whereas LGG elevated GABA levels only in the hippocampus. The changes in neurotransmitter levels were accompanied by the induction of anti-oxidant and anti-inflammatory state. The fact that post-mortem AD patient brain biopsies reveal decreased GABA levels underlines the protective nature of this inhibitory neurotransmitter against AD onset [104]. In the same fashion, hippocampal acetylocholine (Ach) decelerates AD onset. *L. plantarum* MTCC stimulated Ach production in the hippocampus and cerebral cortex and reversed histopathology features in AD Wistar rats. These cellular changes resulted in improvement of behavior and learning skills [105]. To date, only one clinical trial has been performed to investigate the outcomes of probiotic administration to AD patients. In this study, the participants consumed either plain milk or milk enriched with *L. acidophilus*, *L. casei*, *B. bifidum*, and *L. fermentum* for 12 weeks, and the results were evaluated based on the mini-mental state examination (MMSE) scores and the concentration of related plasma biomarkers. The group that consumed probiotics had significantly improved MMSE scores and favorable changes in malondialdehyde and hs-CRP levels, markers of insulin metabolism, and serum triglyceride and very low-density lipoprotein (VLDL) levels. On the other hand, markers of inflammation, oxidative stress, and other lipid profiles remained unchanged [84]. 

Several studies conducted on murine MS models showed that administration of monospecies [106,107], mixtures [108,109], heat-killed strains [110], or genetically modified probiotics [111] provoke specific immunomodulatory effects. Most of the utilized strains switched T cell differentiation towards Tregs and up-regulated the production of the anti-inflammatory cytokine IL-10, while down-regulating the expression of the pro-inflammatory cytokines IL-17 and IL-6, both at the periphery and on site. Overall, blocking of inflammation strengthened the gut barrier and decreased the incidence of autoimmunity, while the activation of Tregs attenuated MS symptoms and progression. These positive results were recapitulated in clinical studies. A probiotic capsule containing *L. acidophilus, L. casei, L. fermentum*, and *B. bifidum* positively affected mental health and disability scores, glucose and lipid metabolism, and inflammation in MS patients [85]. Another study compared changes on the expression of immune related genes in healthy volunteers and MS patients after consumption of probiotics. MS patients who received the probiotic mixture containing *Lactobacilli, Bifidobacteria*, and *Streptococci* strains exhibited decreased expression of CD80 on monocytes and of HLA-DR MFI on dendritic cells. Likewise, the probiotic-treated healthy volunteers had lower expression of the MS risk allele HLA-DQ1. The populations of Th1 and Th17 were reduced in both the controls and MS patients, while memory CD8+ T populations were increased and LAP+ Tregs were decreased exclusively on MS patients and controls, respectively. However, these findings were not correlated with clinical improvement [86]. 

So far, the direct effects of probiotics on Parkinson’s disease-related motor dysfunction have been poorly investigated. Most of the clinical studies investigating probiotic actions in PD have focused on the reduction of its comorbidities, one of which is gut dysfunction that stems, mainly, from enteric neuronal damage and intestinal inflammation. Indeed, Houser et al., confirmed the presence of inflammation biomarkers in PD patients’ stools [112]. In this context, a recent study assessed the anti-inflammatory potential of *L. acidophilus*, *B. bifidum*, *L. reuteri*, and *L. fermentum* on PD patients. The probiotic mixture dampened inflammatory responses by suppressing the expression of IL-1, IL-8, and TNF-α in peripheral blood mononuclear cells (PBMC) and upregulating the expression of transforming growth factor-β (TGF-β) in the same cellular subset [87]. The decrease of gut inflammation could potentially reflect the normalization of gut functionality. In this context, consumption of fermented milk containing multiple probiotic strains and prebiotic fiber was superior to placebo in improving constipation in patients with PD [113]. Furthermore, *L. acidophilus* and *B. infantis* supplementation resolved abdominal pain and bloating in similar levels to trimebutine, a drug commonly used to restore normal bowel function [114]. 

## 5. New Directions in Probiotic Research

Probiotics are thought to exert strain-specific health promoting actions. Indeed, it has been reported that strains of the same species induce differential effects on host physiology. A recent metanalysis of preclinical and clinical data concluded that *Lactobacillus* and *Saccharomyces* treatments exhibit variable results and efficacy on the alleviation of antibiotics-associated diarrhea and IBS symptoms, largely depending on the strain used [115]. Therefore, probiotic research should follow a case-by-case approach when examining the beneficial actions of specific strains on human health. In addition, according to preclinical studies, gender and person-specific traits (such as sexual dimorphism in immune responses and gut microbiota composition) define the success of probiotic supplementation. For instance, administration of *L. reuteri* BM36301 to male and female murine models of aging resulted in reduction of inflammation and in enhancement of skin health exclusively in female mice [40]. Similarly, female Wistar rats that consumed *Lactobacillus farciminis* exhibited reduced gut inflammation and visceral analgesia, while male rats did not experience these beneficial effects [116]. Gender-specific microbiota composition affects the central nervous system (CNS) serotonergic activity, resulting in differential anxiety manifestations in mice. Probiotics also appear to regulate tryptophan availability [117], a serotonin precursor, only in male germ-free mice [118]. Similarly, *L. reuteri* 6475 exerts beneficial effects on bone health of female osteoporotic mice after a mild inflammatory status is induced [119]. 

In an elegant study, Suez et al., recently showed that human microbiota exert a person-specific probiotic colonization resistance profile. Healthy human volunteers that consumed a mixture of 11 commercially-available probiotic strains exhibited differential permissibility to probiotic colonization. Considering differences in absolute abundance of strains in the gut after probiotics administration, participants were clustered into colonization permissive and resistant individuals. The bioinformatic analysis of tissue and mucosal samples acquired by colonoscopy and gastroscopy revealed that probiotic gut colonization is not binary; rather it varies greatly among the permissive volunteers. The person-specific results were attributed to two factors:—permissive individuals had lower levels of probiotic commensals and decreased activity of immune-related pathways in their gut. Particularly, these pathways were linked to innate and adaptive immune responses. Interestingly, both groups of permissive and resistant individuals exhibited a “steady washout” of probiotic strains during the intervention, so stool microbiota is not representative of gut colonization. These findings pave the way for personalized probiotics [120].

In this direction, the first phase of Human Microbiome Project (HMP), which was completed in 2013, managed to characterize a core healthy human skin, gastrointestinal, and urogenital microbiota structure and function by utilizing robust, high through-put sequencing techniques and bioinformatics tools [121,122]. The second phase of the project, the integrative HMP (iHMP), is ongoing and aims at deciphering the role of microbiota in health and disease. Particularly, iHMP investigates the link of microbiota to preterm birth, the onset of IBD, and type-II diabetes. Accumulating evidence reveals the concurrence of microbiota structural and functional imbalances, termed as dysbiosis, with the onset of common, bone [123], skin [124], and nervous system disorders [125]. The host’s genetic makeup could also be considered as a dysbiosis risk factor [126,127]. Genome-wide association studies revealed 58 single nucleotide polymorphisms (SNPs) that are associated with the relative abundance of specific taxa [128] and 33 SNPs were associated with specific microbial pathways [129]. These polymorphisms are located in loci, which are implicated in variations in the vitamin D receptor [130], in immune-related pathways, such as JAK/STAT signaling, chemokine signaling, C-X-C chemokine receptor type-4 (CXCR4) and pattern recognition receptor (PRR) signaling, as well as in melatonin and leptin signaling, inositol phosphate metabolism, gonadotropin-releasing hormone (GnRH) signaling pathway, bile acid synthesis, and sulfide oxidation to sulfate [131]. 

Fecal-Microbiome Transplant (FMT) is gaining momentum in precise microbiota alteration. The first successful application of FMT rehabilitated the gut microbiome post-antibiotic treatment and inhibited *Clostridium difficile* colonization [132]. Notably, a recent study showed that FMT is a more effective approach in post-antibiotics microbiota structural and functional reconstitution than probiotics. *Lactobacillus* probiotic strains inhibit the proliferation of commensals by secreting yet-to be characterized inhibitory compounds [133]. FMT could be applied as an alternative therapy to other non-infectious extraintestinal diseases [134]. In this context, a clinical study has shown that FMT from lean donors to patients with metabolic diseases reversed the pathogenic phenotype [135]. Similarly, Kragsnaes et al., have proposed recently a working protocol to investigate the effects of FMT from healthy donors to people suffering from psoriatic arthritis [136]. Gut microbiota also regulates patients’ response to chemotherapy and immunotherapy. To this aim, the donation of the microbiota of responsive patients to non-responders is expected to increase therapy success [137]. Skin microbiota transplantation could also be useful for alleviating skin conditions [138]. In an attempt to establish a successful protocol for skin microbiome transplantation in patients suffering from skin disorders, Perin et al. managed to successfully transplant forearm microbiota to the back of participants with long-lasting effects being observed in two out of four volunteers [139]. 

Appropriate delivery is a crucial factor for probiotic efficacy. To observe direct effects on skin lesions, probiotics need to be applied topically. A major limitation in this approach is that live probiotics are not compatible to other components of creams, resulting in a rapid organoleptic decline. To combat this limitation, probiotic research has been redirected to the investigation of the properties of cell-free supernatants (CFS) and inactivated strains, termed as “paraprobiotics” [140]. Certain paraprobiotics exert anti-microbial, anti-inflammatory, and anti-aging actions, while being stable in preparations [41,58,59]. Similarly, beneficial outcomes have been reported in preclinical models of bone and CNS pathophysiology after CFS or heat-killed probiotics treatment. In this regard, heat-killed *Lactococcus lactis cremonis* H61 protected senescence-accelerated mice from bone loss and aging skin manifestations [141] and heat-killed *Pediococcus acidilactici* R057 induced immunomodulatory results against a preclinical model of multiple sclerosis [110]. Additionally, several studies have reported the anti-proliferative potential of *Lactobacillus casei* CFS in vitro [142,143]. Konishi et al., managed to isolate and characterize ferrichrome, the active component in this preparation, and compared its action to the established anti-cancer drugs, 5-fluorouracil and cisplatin. Notably, ferrichrome exhibited a better anti-proliferative activity than the anti-cancer drugs, and the authors proposed that this active compound of probiotic origin could be utilized to halt colon carcinoma progression [143]. Importantly, sterile CFS and its isolated components make probiotic supplementation accessible to the vulnerable populations of immunocompromised or immunodeficient individuals, to whom live microorganism consumption is not advised.

Finally, dietary components, such as prebiotic fibers, could enhance the efficiency of probiotic supplementation. The most commonly used prebiotics are non-digestible complex carbohydrates, such as fructooligosaccharides (FOS), galactoologisaccharides (GOS), and inulin. Prebiotics can be utilized as carbon sources for primary or secondary metabolism of specific gut commensals and probiotics [144]. Their beneficial effects on human physiology mainly stem from their ability to induce taxonomic and metabolic shifts in the gut microbial communities. The combinatory administration of prebiotic fibers and probiotic strains, termed as “synbiotics”, most frequently includes mixtures of *Lactobacilli* or *Bifidobacteria* strains admixed with FOS or inulin [145]. However, a limited number of clinical trials have evaluated the effect of synbiotics on human health and disease, and so far, no specific guidelines have been established for their use.

## 6. Conclusions 

Probiotic supplementation aiming at the attenuation of extraintestinal pathological conditions is a promising novel therapeutic approach. However, lack of consistency of the outcomes observed between animal and clinical studies attributed to the differences in gut microbiota composition represents a major hurdle that hinders the clinical application of probiotic strains. Thus, carefully designed protocols for probiotic use that consider strain-, host-, and sex- specificity of their action and optimal route for their administration could maximize their efficacy. In this line, the characterization of the human-microbiome interactome has set the pace for targeted interventions. Whether probiotics or FMT are more potent in microbiota reconstitution is still under debate. Nonetheless, both approaches could potentially benefit people suffering from common disorders, such as osteoporosis, non-healing wounds and autoimmune skin illnesses, mood, behavior, memory, and cognitive impairments. The latest trend in probiotic research, next-generation probiotics, goes beyond traditional terminology and includes microorganisms with beneficial actions that are not members of the commonest probiotic genera, *Lactobacillus* and *Bifidobacteria*. These microorganisms could be utilized in drug discovery, but not for the production of foodstuffs [146]. The significant technological advancements of the last decade have accelerated the understanding of microbiota- and probiotic-induced effects on the host. The translation of this knowledge in clinical practice is expected to flourish in the years to come.

## Figures and Tables

**Table 1:** Summary of clinical trials investigating the beneficial effects of probiotics on bone diseases.

Probiotic Strain	Type of Study	Regimen	Findings	Clinical Outcomes	Reference
Kefir-fermented milk	40 osteoporosis patients participated in a controlled, parallel, double-blind intervention study	Consumption of 1600 mg kefir-fermented milk per day for 6 months	↑serum PTH ↑β-CTX ↑Osteocalcin	Increased femoral neck bone mineral density	[31]
*L. casei* Shirota	537 knee osteoarthritis patients participated in a double-blind, placebo-controlled trial	Oral administration, daily dose of 6 × 10^9^ CFU for 6 months	↓WOMAC ↓VAS score ↓hs-CRP	Inflammation decrease	[32]
*L. casei* Shirota	417 elderly patients with acute distal radius fracture participated in a double-blind, placebo-controlled trial	Oral administration, daily dose of 1.2 × 10^10^ CFU for 6 months	↓DASH score ↓pain VAS ↓CRPS score	Faster fracture rehabilitation	[33]
*L. acidophilus,* *L. casei,* *B. bifidum*	60 RA patients participated in a randomized, double-blind, placebo-controlled trial	One capsule daily for 8 weeks (viable and freeze-dried strains at 2 × 10^9^ CFU/g each)	↓hs-CRP ↓DAS-28 ↓Insulin ↓HOMA-B	Inflammation decrease	[34]
*L. casei* 01	60 female RA patients participated in a randomized double-blind clinical trial	One capsule daily for 8 weeks (10^8^ CFU/capsule)	↓hs-CRP ↓DAS-28 ↓IL-12 ↓TNF-α	Inflammation decrease	[35]

PTH: parathyroid hormone; β-CTX: β C-terminal telopeptide of type I collagen; CFU: colony forming units; WOMAC: Western Ontario and McMaster Universities osteoarthritis index; VAS: visual analogue scale; hs-CRP: highly-sensitive C-reactive protein; DASH: disabilities of the arm, shoulder and hand; CRPS: complex regional pain syndrome; RA: rheumatoid arthritis; DAS-28: disease activity score-28; HOMA-B: homeostasis model of assessment-estimated insulin resistance; IL-12: inteleukin-12; TNF-α: tumor necrosis factor-α (↑: upregulation, ↓: downregulationPage: 4).

**Table 2:** Summary of clinical trials investigating the beneficial effects of probiotics on skin disorders.

Probiotic Strain	Type of Study	Regimen	Findings	Clinical Outcomes	Reference
*B. longum*	66 female volunteers with reactive skin participated in a randomized, double-blind, placebo-controlled trial	Skin application, twice daily for 2 months	↓TEWL	Increased skin hydration and resistance to physical aggression, while limiting skin sensitivity	[41]
*L. brevis* DSM17250	30 volunteers with xerosis symptoms participated in a randomized, placebo-controlled, double blinded pilot study	Skin application, twice daily for 4 weeks	↑Natural skin microbiota ↓DASI ↓TEWL	*L. brevis* supported the proliferation of *S. epidermidis*, resulting in symptom resolution	[43]
*L. rhamnosus* SP1	20 adult volunteers with acne participated in a pilot, randomized, double-blinded, placebo-controlled trial	Consumption of liquid supplement once daily for 12 weeks, 3 × 10^9^ CFU/day	↓IGF-1 ↑FOXO1	Improvement in skin appearance	[47]
*B. infantis* 35624	26 adult psoriasis patients and 36 healthy volunteers participated in a randomized, double-blind, placebo-controlled study	Oral administration, once daily for 6–12 weeks (1 × 10^10^ CFU)	↓CRP ↓TNF-α	Systemic inflammation decrease	[50]
*L. plantarum* IS-10506	22 children with mild and moderate AD participated in a randomized, double-blind, placebo-controlled trial	Oral administration, twice daily for 12 weeks (10^10^ CFU/day)	↓SCORAD ↑FOXP3+/IL-10	Th2 adaptive immune response was suppressed resulting in clinical improvement	[53]
*L. acidophilus* L-92	49 AD patients participated in a double-blind, parallel-group, placebo-controlled study	Oral consumption, once daily for 8 weeks	↓SCORAD ↓eosinophil count ↑TGF-β	Suppression of Th2-dominant inflammation and clinical improvement	[54]
*L. johnsonii* NCC 533	31 AD patients participated in an open-label, multicenter study	Local application, twice daily for 3 weeks	Stable *S. aureus* load ↓SCORAD	*S. aureus* colonization was controlled, followed by local clinical improvement of lesioned skin	[59]
DermaACB	62 consecutive patients with aged skin after fractional CO_2_ laser treatment	Skin application, twice daily for 2 weeks	↓erythema, swelling downtime	Post-operative inflammation signs were reduced	[74]

TEWL: trans-epidermal water loss; DASI: dry skin area and severity index; CFU: colony forming units; IGF-1: insulin-like growth factor-1; FOXO1: forkhead box protein O1; CRP: C-reactive protein; TNF-α: tumor necrosis factor-α; AD: atopic dermatitis; SCORAD: scoring atopic dermatitis; FOXP3+: forkhead box protein P3; IL-10: interleukin-10; TGF-β: transforming growth factor-β (↑: upregulation, ↓: downregulation).

**Table 3:** Summary of clinical trials investigating the effects of probiotics on neuronal health and disease.

Probiotic Strains	Type of Study	Regimen	Findings	Clinical Outcomes	Reference
*L. acidophilus*, *L. casei*, *B. bifidum* (viable and freeze-dried)	40 patients with major depressive disorder participated in a randomized, double-blind, placebo-controlled trial	Once daily (capsule containing 2 × 10^9^ CFU/g of each strain) for 8 weeks	↓BDI, ↓serum insulin ↓HOMA-R, ↓hs-CRP ↑glutathione	Clinical improvement	[75]
*L. casei* strain Shirota	35 chronic fatigue syndrome patients participated in a randomized, double-blind, placebo-controlled pilot study	Oral consumption, three times daily for 8 weeks (8 × 10^9^ CFU)	↑*Lactobacillus*, *Bifidobacteria* gut populations, ↓anxiety symptoms	Reduced anxiety symptoms	[76]
*L. helveticus* R0052 and *B. longum* R0175	55 healthy volunteers	Oral consumption, one stick per day (3 × 10^9^ CFU/stick)	↑HSCL-90 scale, ↑HADS, ↓UFC	Psychological distress reduction	[77]
*B. longum 1714*	22 healthy male volunteers participated in a repeated measures, placebo-controlled study	Oral administration, one stick (1 × 10^9^ CFU) each morning for 4 weeks	Changes in brain electrophysiology (EEG profile)	Dampened stress responses and improved memory	[78]
*L. casei* strain Shirota YIT 9029	49 healthy students participated in a double-blind, placebo-controlled, and parallel-group clinical trial	Daily consumption, 100 mL/day (1 × 10^11^ CFU/100 mL) for 8 weeks	↓VAS, ↓GSRS, ↓abdominal dysfunction score, ↑observed species ↑phylogenetic diversity	The onset of stress-associated abdominal symptoms was decelerated	[79]
*B. bifidum* R0071	655 healthy students participated in a randomized, double-blind, placebo-controlled study	One capsule *per os* (3 × 10^9^ CFU) a day for 6 weeks	↓BSFS score, ↓self-reported stress	The onset of stress-associated abdominal symptoms was decelerated	[80]
*B. bifidum* R0071	581 students participated in a randomized, double-blind, placebo-controlled study	One capsule *per os* (3 × 10^9^ CFU) a day for 7 weeks	↑Proportion of healthy days, ↓cold/flu episodes	The placebo group were less likely to report a sick day	[81]
*L. brevis* SBC8803 (SBL88™)	17 males with poor quality of sleep participated in a non-randomized, double blind, placebo-controlled and crossover pilot study	Oral administration, once daily for 10 days (25 mg/day)	↑delta power value (μV^2^/min)	Sleep quality was increased	[82]
*L. rhamnosus* HN001	423 women at 14–16 weeks gestation participated in a randomized double-blind, placebo-controlled trial	Oral administration, 6 × 10^9^ CFU/day from enrollment to 6 months post-partum	↓EPDS, ↓STAI6	Lower post-partum depression and anxiety scores	[83]
*L. acidophilus, L. casei, B. bifidum, L. fermentum*	60 patients with AD participated in a randomized, double-blind and controlled trial	Oral consumption of 200 mL/day of fermented milk (2 × 10^9^ CFU/mL of each probiotic) for 12 weeks	↑MMSE, ↓MDA, ↓hs-CRP ↓HOMA-B, ↑QUICKI ↓TG, ↓VLDL	Improved cognitive function	[84]
*L. acidophilus, L. casei, B. bifidum, L. fermentum*	60 MS patients participated in a randomized, double-blind, placebo-controlled trial	Oral administration, once daily for 12 weeks (capsules containing 2 × 10^9^ CFU/g of each strain)	↓EDSS, ↓BDI, ↓DASS, ↓hs-CRP, ↓serum insulin ↓HOMA-R	Mental health improvement	[85]
*L. paracasei* 24734, *L. plantarum* 24730, *L. acidophilus* 24735, *L. delbruckei bulgaricus* 24734, *B. longum* 24736, *B. infantis* 24737, *B. breve* 24732, *S. thermophilus* 2473	9 MS patients and 13 healthy volunteers	Oral administration, twice daily for 2 months (3.6 × 10^9^ CFU/day)	↓KEGG pathway abundance ↑ CD8+T cells, ↑IL-10RA, ↑LILRB2, ↑CYBB ↓MALT1, ↓LGALS3	Reduced dysbiosis and inflammation, a synergistic effect with current medication was observed and the expression of MS-risk alleles was decreased	[86]
*L. acidophilus,* *B. bifidum, L. reuteri,* *L. fermentum*	50 patients with PD participated in a randomized, double-blind, placebo-controlled pilot study	One capsule *per os* a day (8 × 10^9^ CFU) for 12 weeks	↓IL-1, ↓IL-8, ↓TNF-α, ↑TGF-β, ↑PPAR-γ	N/A	[87]

CFU: colony forming units; BDI: Beck depression inventory; HOMA-R: homeostasis model of assessment-estimated insulin resistance; hs-CRP: high-sensitivity C-reactive protein; HSCL-90: Hopkins symptom checklist; HADS: hospital anxiety and depression scale; UFC: urinary free cortisol; EEG: electroencephalography; VAS: visual analogue scale; GSRS: gastrointestinal symptom rating scale; BSFS: Bristol stool form scale; EPDS: Edinburgh postnatal depression scale; STAI: state trait anxiety inventory; AD: Alzheimer’s disease; MMSE: mini mental state examination; MDA: malonaldehyde; MS: multiple sclerosis HOMA-B: homeostasis model assessment of β-cell function; QUICKI: quantitative insulin sensitivity check index; TG: triglyceride; VLDL: very low density lipoprotein; EDSS: expanded disability status scale; DASS: depression anxiety and stress scale; KEGG: Kyoto encyclopedia of genes and genomes; IL-10RA: interleukin-10 receptor; LILRB2: leukocyte immunoglobulin-like receptor subfamily B member 2; CYBB: cytochrome b beta chain; MALT1: mucosa-associated lymphoid tissue lymphoma translocation protein 1; LGALS3: galectin-3; PD: Parkinson’s disease; IL-1: interleukin-1; IL-8: interleukin-8; TNF-α: tumor necrosis factor-α; TGF-β: tumor growth factor-β; PPAR-γ: peroxisome proliferator-activated receptor gamma; N/A: Not Available (↑: upregulation, ↓: downregulation).

## References

[1] Food and Agriculture Organization of the United Nations/World Health Organization (FAO/WHO) (2002). Evaluation of Health and Nutritional Properties of Powder Milk and Live Lactic Acid Bacteria. www.fao.org/3/a-a0512e.pdf.

[2] Wilkins T., Sequoia J. (2017). Probiotics for Gastrointestinal Conditions: A Summary of the Evidence. Am. Fam. Physician.

[3] Yousefi B., Eslami M., Ghasemian A., Kokhaei P., Salek Farrokhi A., Darabi N. (2018). Probiotics importance and their immunomodulatory properties. J. Cell. Physiol..

[4] Plaza-Diaz J., Ruiz-Ojeda F.J., Gil-Campos M., Gil A. (2019). Mechanisms of Action of Probiotics. Adv. Nutr..

[5] Saxami G., Karapetsas A., Chondrou P., Vasiliadis S., Lamprianidou E., Kotsianidis I., Ypsilantis P., Botaitis S., Simopoulos C., Galanis A. (2017). Potentially probiotic Lactobacillus strains with anti-proliferative activity induce cytokine/chemokine production and neutrophil recruitment in mice. Benef. Microbes.

[6] Chondrou P., Karapetsas A., Kiousi D.E., Tsela D., Tiptiri-Kourpeti A., Anestopoulos I., Kotsianidis I., Bezirtzoglou E., Pappa A., Galanis A. (2018). Lactobacillus paracasei K5 displays adhesion, anti-proliferative activity and apoptotic effects in human colon cancer cells. Benef. Microbes.

[7] Luczynski P., McVey Neufeld K.A., Oriach C.S., Clarke G., Dinan T.G., Cryan J.F. (2016). Growing up in a Bubble: Using Germ-Free Animals to Assess the Influence of the Gut Microbiota on Brain and Behavior. Int. J. Neuropsychopharmacol..

[8] Abrahamsson T.R., Jakobsson H.E., Andersson A.F., Björkstén B., Engstrand L., Jenmalm M.C. (2014). Low gut microbiota diversity in early infancy precedes asthma at school age. Clin. Exp. Allergy.

[9] Thushara R.M., Gangadaran S., Solati Z., Moghadasian M.H. (2016). Cardiovascular benefits of probiotics: A review of experimental and clinical studies. Food Funct..

[10] Aggarwal J., Swami G., Kumar M. (2013). Probiotics and their Effects on Metabolic Diseases: An Update. J. Clin. Diagn. Res..

[11] Collins F.L., Rios-Arce N.D., Schepper J.D., Parameswaran N., McCabe L.R. (2017). The Potential of Probiotics as a Therapy for Osteoporosis. Microbiol. Spectr..

[12] Roudsari M.R., Karimi R., Sohrabvandi S., Mortazavian A.M. (2013). Health Effects of Probiotics on the Skin. Crit. Rev. Food. Sci. Nutr..

[13] Slyepchenko A., Carvalho A.F., Cha D.S., Kasper S., McIntyre R.S. (2014). Gut emotions-mechanisms of action of probiotics as novel therapeutic targets for depression and anxiety disorders. CNS Neurol. Disord. Drug Targets.

[14] Westfall S., Lomis N., Kahouli I., Dia S.Y., Singh S.P., Prakash S. (2017). Microbiome, probiotics and neurodegenerative diseases: Deciphering the gut brain axis. Cell Mol. Life Sci..

[15] Islam S.U. (2016). Clinical Uses of Probiotics. Medicine.

[16] Kim Y.G., Lee C.K., Nah S.S., Mun S.H., Yoo B., Moon H.B. (2007). Human CD4+CD25+ regulatory T cells inhibit the differentiation of osteoclasts from peripheral blood mononuclear cells. Biochem. Biophys. Res. Commun..

[17] Weitzmann M.N., Pacifici R. (2006). Estrogen deficiency and bone loss: An inflammatory tale. J. Clin. Investig..

[18] Narva M., Halleen J., Väänänen K., Korpela R. (2004). Effects of *Lactobacillus helveticus* fermented milk on bone cells in vitro. Life Sci..

[19] Narva M., Rissanen J., Halleen J., Vapaatalo H., Väänänen K., Korpela R. (2007). Effects of bioactive peptide, valyl-prolyl-proline (VPP), and lactobacillus helveticus fermented milk containing VPP on bone loss in ovariectomized rats. Ann. Nutr. MeTable.

[20] Ohlsson C., Engdahl C., Fåk F., Andersson A., Windahl S.H., Farman H.H., Movérare-Skrtic S., Islander U., Sjögren K. (2014). Probiotics protect mice from ovariectomy-induced cortical bone loss. PLoS ONE.

[21] Tomofuji T., Ekuni D., Azuma T., Irie K., Endo Y., Yamamoto T., Ishikado A., Sato T., Harada K., Suido H. (2012). Supplementation of broccoli or Bifidobacterium longum-fermented broccoli suppresses serum lipid peroxidation and osteoclast differentiation on alveolar bone surface in rats fed a high-cholesterol diet. Nutr. Res..

[22] Britton R.A., Irwin R., Quach D., Schaefer L., Zhang J., Lee T., Parameswaran N., McCabe L.R. (2014). Probiotic *L. reuteri* Treatment Prevents Bone Loss in a Menopausal Ovariectomized Mouse Model. J. Cell. Physiol..

[23] Li J.Y., Chassaing B., Tyagi A.M., Vaccaro C., Luo T., Adams J., Darby T.M., Weitzmann M.N., Mulle J.G., Gewirtz A.T. (2016). Sex steroid deficiency-associated bone loss is microbiota dependent and prevented by probiotics. J. Clin. Investig..

[24] Chiang S.S., Pan T.M. (2011). Antiosteoporotic effects of Lactobacillus- fermented soy skim milk on bone mineral density and the microstructure of femoral bone in ovariectomized mice. J. Agric. Food Chem..

[25] Rodrigues F.C., Castro A.S.B., Rodrigues V.C., Fernandes S.A., Fontes E.A.F., de Oliveira T.T., Martino H.S.D., de Luces Fortes Ferreira C.L. (2012). Yacon flour and Bifidobacterium longum modulate bone health in rats. J. Med. Food.

[26] Morrison D.J., Preston T. (2016). Formation of short chain fatty acids by the gut microbiota and their impact on human metabolism. Gut Microbes.

[27] Gonçalves P., Araújo J., Di Santo J.P. (2018). A Cross-Talk Between Microbiota-Derived Short-Chain Fatty Acids and the Host Mucosal Immune System Regulates Intestinal Homeostasis and Inflammatory Bowel Disease. Inflamm. Bowel Dis..

[28] Lucas S., Omata Y., Hofmann J., Böttcher M., Iljazovic A., Sarter K., Albrecht O., Schulz O., Krishnacoumar B., Krönke G. (2018). Short-chain fatty acids regulate systemic bone mass and protect from pathological bone loss. Nat. Commun..

[29] Jafarnejad S., Djafarian K., Fazeli M.R., Yekaninejad M.S., Rostamian A., Keshavarz S.A. (2017). Effects of a Multispecies Probiotic Supplement on Bone Health in Osteopenic Postmenopausal Women: A Randomized, Double-blind, Controlled Trial. J. Am. Coll. Nutr..

[30] Takimoto T., Hatanaka M., Hoshino T., Takara T., Tanaka K., Shimizu A., Morita H., Nakamura T. (2018). Effect of Bacillus subtilis C-3102 on bone mineral density in healthy postmenopausal Japanese women: A randomized, placebo-controlled, double-blind clinical trial. Biosci. Microbiota Food Health.

[31] Tu M.Y., Chen H.L., Tung Y.T., Kao C.C., Hu F.C., Chen C.M. (2015). Short-Term Effects of Kefir-Fermented Milk Consumption on Bone Mineral Density and Bone Metabolism in a Randomized Clinical Trial of Osteoporotic Patients. PLoS ONE.

[32] Lei M., Guo C., Wang D., Zhang C., Hua L. (2017). The effect of probiotic Lactobacillus casei Shirota on knee osteoarthritis: A randomised double-blind, placebo-controlled clinical trial. Benef. Microbes.

[33] Lei M., Hua L.M., Wang D.W. (2016). The effect of probiotic treatment on elderly patients with distal radius fracture: A prospective double-blind, placebo-controlled randomised clinical trial. Benef. Microbes.

[34] Zamani B., Golkar H.R., Farshbaf S., Emadi-Baygi M., Tajabadi-Ebrahimi M., Jafari P., Akhavan R., Taghizadeh M., Memarzadeh M.R., Asemi Z. (2016). Clinical and metabolic response to probiotic supplementation in patients with rheumatoid arthritis: A randomized, double-blind, placebo-controlled trial. Int. J. Rheum. Dis..

[35] Alipour B., Homayouni-Rad A., Vaghef-Mehrabany E., Sharif S.K., Vaghef-Mehrabany L., Asghari-Jafarabadi M., Nakhjavani M.R., Mohtadi-Nia J. (2014). Effects of Lactobacillus casei supplementation on disease activity and inflammatory cytokines in rheumatoid arthritis patients: A randomized double-blind clinical trial. Int. J. Rheum. Dis..

[36] Yamashita M., Matsumoto K., Endo T., Endo T., Ukibe K., Hosoya T., Matsubara Y., Nakagawa H., Sakai F., Miyazaki T. (2017). Preventive Effect of *Lactobacillus helveticus* SBT2171 on Collagen-Induced Arthritis in Mice. Front. Microbiol..

[37] Lee S.Y., Lee S.H., Jhun J., Seo H.B., Jung K.A., Yang C.W., Park S.H., Cho M.L. (2018). A Combination with Probiotic Complex, Zinc, and Coenzyme Q10 Attenuates Autoimmune Arthritis by Regulation of Th17/Treg Balance. J. Med. Food.

[38] Arck P.C., Slominski A., Theoharides T.C., Peters E.M., Paus R. (2006). Neuroimmunology of stress: Skin takes center stage. J. Invest. Dermatol..

[39] Choi J.E., Di Nardo A. (2018). Skin neurogenic inflammation. Semin. Immunopathol..

[40] Lee J., Yang W., Hostetler A., Schultz N., Suckow M.A., Stewart K.L., Kim D.D., Kim H.S. (2016). Characterization of the anti-inflammatory Lactobacillus reuteri BM36301 and its probiotic benefits on aged mice. BMC Microbiol..

[41] Guéniche A., Bastien P., Ovigne J.M., Kermici M., Courchay G., Chevalier V., Breton L., Castiel-Higounenc I. (2009). Bifidobacterium longum lysate, a new ingredient for reactive skin. Exp. Dermatol..

[42] Wang Y., Kao M.S., Yu J., Huang S., Marito S., Gallo R.L., Huang C.M. (2016). A Precision Microbiome Approach Using Sucrose for Selective Augmentation of Staphylococcus epidermidis Fermentation against Propionibacterium acnes. Int. J. Mol. Sci..

[43] Holz C., Benning J., Schaudt M., Heilmann A., Schultchen J., Goelling D., Lang C. (2017). Novel bioactive from Lactobacillus brevis DSM17250 to stimulate the growth of Staphylococcus epidermidis: A pilot study. Benef. Microbes.

[44] Al-Ghazzewi F.H., Tester R.F. (2010). Effect of konjac glucomannan hydrolysates and probiotics on the growth of the skin bacterium Propionibacterium acnes in vitro. Int. J. Cosmet. Sci..

[45] Oh S., Kim S.H., Ko Y., Sim J.H., Kim K.S., Lee S.H., Park S., Kim Y.J. (2006). Effect of bacteriocin produced by Lactococcus sp. HY 449 on skin-inflammatory bacteria. Food Chem. Toxicol..

[46] Manzhalii E., Hornuss D., Stremmel W. (2016). Intestinal-borne dermatoses significantly improved by oral application of Escherichia coli Nissle 1917. World J. Gastroenterol..

[47] Fabbrocini G., Bertona M., Picazo O., Pareja-Galeano H., Emanuele E. (2016). Supplementation with Lactobacillus rhamnosus SP1 normalises skin expression of genes implicated in insulin signalling and improves adult acne. Benef. Microbes.

[48] Hawkes J.E., Chan T.C., Krueger J.G. (2017). Psoriasis pathogenesis and the development of novel targeted immune therapies. J. Allergy Clin. Immunol..

[49] Chen Y.H., Wu C.S., Chao Y.H., Lin C.C., Tsai H.Y., Li Y.R., Chen Y.Z., Tsai W.H., Chen Y.K. (2017). Lactobacillus pentosus GMNL-77 inhibits skin lesions in imiquimod-induced psoriasis-like mice. J. Food Drug Anal..

[50] Groeger D., O’Mahony L., Murphy E.F., Bourke J.F., Dinan T.G., Kiely B., Shanahan F., Quigley E.M. (2013). Bifidobacterium infantis 35624 modulates host inflammatory processes beyond the gut. Gut Microbes.

[51] Satta R., Pes G.M., Rocchi C., Pes M.C., Dore M.P. (2018). Is probiotic use beneficial for skin lesions in patients with inflammatory bowel disease?. J. Dermatolog. Treat..

[52] Kim J.A., Kim S.H., Kim I.S., Yu D.Y., Kim S.C., Lee S.H., Lee S.S., Yun C.H., Choi I.S., Cho K.K. (2018). Anti-Inflammatory Effects of a Mixture of Lactic Acid Bacteria and Sodium Butyrate in Atopic Dermatitis Murine Model. J. Med. Food.

[53] Prakoeswa C.R.S., Herwanto N., Prameswari R., Astari L., Sawitri S., Hidayati A.N., Indramaya D.M., Kusumowidagdo E.R., Surono I.S. (2017). Lactobacillus plantarum IS-10506 supplementation reduced SCORAD in children with atopic dermatitis. Benef. Microbes.

[54] Inoue Y., Kambara T., Murata N., Komori-Yamaguchi J., Matsukura S., Takahashi Y., Ikezawa Z., Aihara M. (2014). Effects of oral administration of Lactobacillus acidophilus L-92 on the symptoms and serum cytokines of atopic dermatitis in Japanese adults: A double-blind, randomized, clinical trial. Int. Arch. Allergy Immunol..

[55] Holowacz S., Blondeau C., Guinobert I., Guilbot A., Hidalgo S., Bisson J.F. (2018). Lactobacillus salivarius LA307 and Lactobacillus rhamnosus LA305 attenuate skin inflammation in mice. Benef. Microbes.

[56] Choi W.J., Konkit M., Kim Y., Kim M.K., Kim W. (2016). Oral administration of Lactococcus chungangensis inhibits 2,4-dinitrochlorobenzene-induced atopic-like dermatitis in NC/Nga mice. J. Dairy Sci..

[57] Lee S.H., Yoon J.M., Kim Y.H., Jeong D.G., Park S., Kang D.J. (2016). Therapeutic effect of tyndallized Lactobacillus rhamnosus IDCC 3201 on atopic dermatitis mediated by down-regulation of immunoglobulin E in NC/Nga mice. Microbiol. Immunol..

[58] Rosignoli C., Thibaut de Ménonville S., Orfila D., Béal M., Bertino B., Aubert J., Mercenier A., Piwnica D. (2018). A topical treatment containing heat-treated Lactobacillus johnsonii NCC 533 reduces Staphylococcus aureus adhesion and induces antimicrobial peptide expression in an in vitro reconstructed human epidermis model. Exp. Dermatol..

[59] Blanchet-Réthoré S., Bourdès V., Mercenier A., Haddar C.H., Verhoeven P.O., Andres P. (2017). Effect of a lotion containing the heat-treated probiotic strain Lactobacillus johnsonii NCC 533 on Staphylococcus aureus colonization in atopic dermatitis. Clin. Cosmet. Investig. Dermatol..

[60] Jones M., Ganopolsky J.G., Labbé A., Gilardino M., Wahl C., Martoni C., Prakash S. (2012). Novel nitric oxide producing probiotic wound healing patch: Preparation and in vivo analysis in a New Zealand white rabbit model of ischaemic and infected wounds. Int. Wound J..

[61] Mohammedsaeed W., Cruickshank S., McBain A.J., O’Neill C.A. (2015). Lactobacillus rhamnosus GG lysate increases reepithelialization of keratinocyte scratch assays by promoting migration. Sci. Rep..

[62] Sultana R., McBain A.J., O’Neill C.A. (2013). Strain-dependent augmentation of tight-junction barrier function in human primary epidermal keratinocytes by Lactobacillus and Bifidobacterium lysates. Appl. Environ. Microbiol..

[63] Lopes E.G., Moreira D.A., Gullón P., Gullón B., Cardelle-Cobas A., Tavaria F.K. (2017). Topical application of probiotics in skin: Adhesion, antimicrobial and antibiofilm in vitro assays. J. Appl. Microbiol..

[64] Mohammedsaeed W., McBain A.J., Cruickshank S.M., O’Neill C.A. (2014). Lactobacillus rhamnosus GG inhibits the toxic effects of Staphylococcus aureus on epidermal keratinocytes. Appl. Environ. Microbiol..

[65] Iwase T., Uehara Y., Shinji H., Tajima A., Seo H., Takada K., Agata T., Mizunoe Y. (2010). Staphylococcus epidermidis Esp inhibits Staphylococcus aureus biofilm formation and nasal colonization. Nature.

[66] Nakatsuji T., Chen T.H., Narala S., Chun K.A., Two A.M., Yun T., Shafiq F., Kotol P.F., Bouslimani A., Melnik A.V. (2017). Antimicrobials from human skin commensal bacteria protect against Staphylococcus aureus and are deficient in atopic dermatitis. Sci. Transl. Med..

[67] Sharma D., Kober M.M., Bowe W.P. (2016). Anti-Aging Effects of Probiotics. J. Drugs Dermatol..

[68] Im A.R., Kim H.S., Hyun J.W., Chae S. (2016). Potential for tyndalized Lactobacillus acidophilus as an effective component in moisturizing skin and anti-wrinkle products. Exp. Ther. Med..

[69] Satoh T., Murata M., Iwabuchi N., Odamaki T., Wakabayashi H., Yamauchi K., Abe F., Xiao J.Z. (2015). Effect of Bifidobacterium breve B-3 on skin photoaging induced by chronic UV irradiation in mice. Benef. Microbes.

[70] Bouilly-Gauthier C., Maubert J.Y., Duteil L., Queille-Roussel C., Piccardi N., Montastier C., Manissier P., Pierard G., Ortonne J.P. (2010). Clinical evidence of benefits of a dietary supplement containing probiotic and carotenoids on ultraviolet-induced skin damage. Br. J. Dermatol..

[71] Mahé Y.F., Martin R., Aubert L., Billoni N., Collin C., Pruche F., Bastien P., Drost S.S., Lane A.T., Meybeck A. (2006). Induction of the skin endogenous protective mitochondrial MnSOD by Vitreoscilla filiformis extract. Int. J. Cosmet. Sci..

[72] Park M.J., Bae Y.S. (2016). Fermented acanthopanax koreanum root extract reduces UVB- and H2O2-induced senescence in human skin fibroblast cells. J. Microbiol. Biotechnol..

[73] Weinstein C., Ramirez O.M., Pozner J.N. (1997). Postoperative care following CO2 laser resurfacing: Avoiding pitfalls. Plast. Reconstr. Surg..

[74] Zoccali G., Cinque B., La Torre C., Lombardi F., Palumbo P., Romano L., Mattei A., Orsini G., Cifone M.G., Giuliani M. (2016). Improving the outcome of fractional CO_2_ laser resurfacing using a probiotic skin cream: Preliminary clinical evaluation. Lasers Med. Sci..

[75] Akkasheh G., Kashani-Poor Z., Tajabadi-Ebrahimi M., Jafari P., Akbari H., Taghizadeh M., Memarzadeh M.R., Asemi Z., Esmaillzadeh A. (2016). Clinical and metabolic response to probiotic administration in patients with major depressive disorder: A randomized, double-blind, placebo-controlled trial. Nutrition.

[76] Rao A.V., Bested A.C., Beaulne T.M., Katzman M.A., Iorio C., Berardi J.M., Logan A.C. (2009). A randomized, double-blind, placebo-controlled pilot study of a probiotic in emotional symptoms of chronic fatigue syndrome. Gut Pathog..

[77] Messaoudi M., Violle N., Bisson J., Desor D., Javelot H., Rougeot C. (2011). Beneficial psychological effects of a probiotic formulation (Lactobacillus helveticus R0052 and Bifidobacterium longum R0175) in healthy human volunteers. Gut Microbes.

[78] Allen A.P., Hutch W., Borre Y.E., Kennedy P.J., Temko A., Boylan G., Murphy E., Cryan J.F., Dinan T.G., Clarke G. (2016). Bifidobacterium longum 1714 as a translational psychobiotic: Modulation of stress, electrophysiology and neurocognition in healthy volunteers. Transl. Psychiatry.

[79] Kato-Kataoka A., Nishida K., Takada M., Kawai M., Kikuchi-Hayakawa H., Suda K., Ishikawa H., Gondo Y., Shimizu K., Matsuki T. (2016). Fermented milk containing Lactobacillus casei strain Shirota preserves the diversity of the gut microbiota and relieves abdominal dysfunction in healthy medical students exposed to academic stress. Appl. Environ. Microbiol..

[80] Culpepper T., Christman M.C., Nieves C., Specht G.J., Rowe C.C., Spaiser S.J., Ford A.L., Dahl W.J., Girard S.A., Langkamp-Henken B. (2016). Bifidobacterium bifidum R0071 decreases stress-associated diarrhoea-related symptoms and self-reported stress: A secondary analysis of a randomised trial. Benef. Microbes.

[81] Langkamp-Henken B., Rowe C.C., Ford A.L., Christman M.C., Nieves C., Khouri L., Specht G.J., Girard S.A., Spaiser S.J., Dahl W.J. (2015). Bifidobacterium bifidum R0071 results in a greater proportion of healthy days and a lower percentage of academically stressed students reporting a day of cold/flu: A randomised, double-blind, placebo-controlled study. Br. J. Nutr..

[82] Nakakita Y., Tsuchimoto N., Takata Y., Nakamura T. (2016). Effect of dietary heat-killed Lactobacillus brevis SBC8803 (SBL88™) on sleep: A non-randomised, double blind, placebo-controlled, and crossover pilot study. Benef. Microbes.

[83] Slykerman R.F., Hood F., Wickens K., Thompson J.M.D., Barthow C., Murphy R., Kang J., Rowden J., Stone P., Crane J. (2017). Effect of Lactobacillus rhamnosus HN001 in Pregnancy on Postpartum Symptoms of Depression and Anxiety: A Randomised Double-blind Placebo-controlled Trial. EBioMedicine.

[84] Akbari E., Asemi Z., Daneshvar Kakhaki R., Bahmani F., Kouchaki E., Tamtaji O.R., Hamidi G.A., Salami M. (2016). Effect of Probiotic Supplementation on Cognitive Function and Metabolic Status in Alzheimer’s Disease: A Randomized, Double-Blind and Controlled Trial. Front. Aging Neurosci..

[85] Kouchaki E., Tamtaji O.R., Salami M., Bahmani F., Daneshvar Kakhaki R., Akbari E., Tajabadi-Ebrahimi M., Jafari P., Asemi Z. (2017). Clinical and metabolic response to probiotic supplementation in patients with multiple sclerosis: A randomized, double-blind, placebo-controlled trial. Clin. Nutr..

[86] Tankou S.K., Regev K., Healy B.C., Tjon E., Laghi L., Cox L.M., Kivisäkk P., Pierre I.V., Lokhande H., Gandhi R. (2018). A probiotic modulates the microbiome and immunity in multiple sclerosis. Ann. Neurol..

[87] Borzabadi S., Oryan S., Eidi A., Aghadavod E., Daneshvar Kakhaki R., Tamtaji O.R., Taghizadeh M., Asemi Z. (2018). The Effects of Probiotic Supplementation on Gene Expression Related to Inflammation, Insulin and Lipid in Patients with Parkinson’s Disease: A Randomized, Double-blind, Placebo Controlled Trial. Arch. Iran Med..

[88] Gareau M.G., Jury J., MacQueen G., Sherman P.M., Perdue M.H. (2007). Probiotic treatment of rat pups normalises corticosterone release and ameliorates colonic dysfunction induced by maternal separation. Gut.

[89] Moya-Pérez A., Perez-Villalba A., Benítez-Páez A., Campillo I., Sanz Y. (2017). Bifidobacterium CECT 7765 modulates early stress-induced immune, neuroendocrine and behavioral alterations in mice. Brain Behav. Immun..

[90] Liang S., Wang T., Hu X., Luo J., Li W., Wu X., Duan Y., Jin F. (2015). Administration of *Lactobacillus helveticus* NS8 improves behavioral, cognitive, and biochemical aberrations caused by chronic restraint stress. Neuroscience.

[91] Savignac H.M., Kiely B., Dinan T.G., Cryan J.F. (2014). Bifidobacteria exert strain-specific effects on stress-related behavior and physiology in BALB/c mice. Neurogastroenterol..

[92] Savignac H.M., Tramullas M., Kiely B., Dinan T.G., Cryan J.F. (2015). Bifidobacteria modulate cognitive processes in an anxious mouse strain. Behav. Brain Res..

[93] Kantak P.A., Bobrow D.N., Nyby J.G. (2014). Obsessive-compulsive-like behaviors in house mice are attenuated by a probiotic (Lactobacillus rhamnosus GG). Behav. Pharmacol..

[94] Tillisch K., Labus J., Kilpatrick L., Jiang Z., Stains J., Ebrat B., Guyonnet D., Legrain-Raspaud S., Trotin B., Naliboff B. (2013). Consumption of fermented milk product with probiotic modulates brain activity. Gastroenterology.

[95] Clark A., Mach N. (2016). Exercise-induced stress behavior, gut-microbiota-brain axis and diet: A systematic review for athletes. J. Int. Soc. Sports Nutr..

[96] Kaelberer M.M., Buchanan K.L., Klein M.E., Barth B.B., Montoya M.M., Shen X., Bohórquez D.V. (2018). A gut-brain neural circuit for nutrient sensory transduction. Science.

[97] McKernan D.P., Fitzgerald P., Dinan T.G., Cryan J.F. (2010). The probiotic Bifidobacterium infantis 35624 displays visceral antinociceptive effects in the rat. Neurogastroenterol. Motil..

[98] Rousseaux C., Thuru X., Gelot A., Barnich N., Neut C., Dubuquoy L., Dubuquoy C., Merour E., Geboes K., Chamaillard M. (2007). Lactobacillus acidophilus modulates intestinal pain and induces opioid and cannabinoid receptors. Nat. Med..

[99] Athari Nik Azm S., Djazayeri A., Safa M., Azami K., Ahmadvand B., Sabbaghziarani F., Sharifzadeh M., Vafa M. (2018). Lactobacillus and Bifidobacterium Ameliorate Memory and Learning Deficits and oxidative stress in Aβ (1-42) Injected Rats. Appl. Physiol. Nutr. MeTable.

[100] Bonfili L., Cecarini V., Cuccioloni M., Angeletti M., Berardi S., Scarpona S., Rossi G., Eleuteri A.M. (2018). LAB51 Probiotic Formulation Activates SIRT1 Pathway Promoting Antioxidant and Neuroprotective Effects in an AD Mouse Model. Mol. Neurobiol..

[101] Cheng Y., Takeuchi H., Sonobe Y., Jin S., Wang Y., Horiuchi H., Parajuli B., Kawanokuchi J., Mizuno T., Suzumura A. (2014). Sirtuin 1 attenuates oxidative stress via upregulation of superoxide dismutase 2 and catalase in astrocytes. J. Neuroimmunol..

[102] Kobayashi Y., Sugahara H., Shimada K., Mitsuyama E., Kuhara T., Yasuoka A., Kondo T., Abe K., Jin-zhong Xiao J. (2017). Therapeutic potential of Bifidobacterium breve strain A1 for preventing cognitive impairment in Alzheimer’s disease. Sci. Rep..

[103] Bhattacharjee S., Lukiw W.J. (2013). Alzheimer’s disease and the microbiome. Front. Cell. Neurosci..

[104] Divyashri G., Krishna G., Muralidhara, Prapulla S.G. (2015). Probiotic attributes, antioxidant, anti-inflammatory and neuromodulatory effects of Enterococcus faecium CFR 3003: In vitro and in vivo evidence. J. Med. Microbiol..

[105] Nimgampalle M., Kuna Y. (2017). Anti-Alzheimer Properties of Probiotic, Lactobacillus plantarum MTCC 1325 in Alzheimer’s Disease induced Albino Rats. J. Clin. Diagn. Res..

[106] Secher T., Kassem S., Benamar M., Bernard I., Boury M., Barreau F., Oswald E., Saoudi A. (2017). Oral Administration of the Probiotic Strain Escherichia coli Nissle 1917 Reduces Susceptibility to Neuroinflammation and Repairs Experimental Autoimmune Encephalomyelitis-Induced Intestinal Barrier Dysfunction. Front. Immunol..

[107] Yamashita M., Ukibe K., Matsubara Y., Hosoya T., Sakai F., Kon S., Arima Y., Murakami M., Nakagawa H., Miyazaki T. (2018). Lactobacillus helveticus SBT2171 Attenuates Experimental Autoimmune Encephalomyelitis in Mice. Front. Microbiol..

[108] Kwon H.K., Kim G.C., Kim Y., Hwang W., Jash A., Sahoo A., Kim J.E., Nam J.H., Im S.H. (2013). Amelioration of experimental autoimmune encephalomyelitis by probiotic mixture is mediated by a shift in T helper cell immune response. Clin. Immunol..

[109] Salehipour Z., Haghmorad D., Sankian M., Rastin M., Nosratabadi R., Soltan Dallal M.M., Tabasi N., Khazaee M., Nasiraii L.R., Mahmoudi M. (2017). Bifidobacterium animalis in combination with human origin of Lactobacillus plantarum ameliorate neuroinflammation in experimental model of multiple sclerosis by altering CD4+ T cell subset balance. Biomed. Pharmacother..

[110] Takata K., Kinoshita M., Okuno T., Moriya M., Kohda T., Honorat J.A., Sugimoto T., Kumanogoh A., Kayama H., Takeda K. (2011). The lactic acid bacterium Pediococcus acidilactici suppresses autoimmune encephalomyelitis by inducing IL-10-producing regulatory T cells. PLoS ONE.

[111] Rezende R.M., Oliveira R.P., Medeiros S.R., Gomes-Santos A.C., Alves A.C., Loli F.G., Guimarães M.A., Amaral S.S., da Cunha A.P., Weiner H.L. (2013). Hsp65-producing Lactococcus lactis prevents experimental autoimmune encephalomyelitis in mice by inducing CD4+LAP+ regulatory T cells. J. Autoimmun..

[112] Houser M.C., Chang J., Factor S.A., Molho E.S., Zabetian C.P., Hill-Burns E.M., Payami H., Hertzberg V.S., Tansey M.G. (2018). Stool immune profiles evince gastrointestinal inflammation in Parkinson’s disease. Mov. Disord..

[113] Barichella M., Pacchetti C., Bolliri C., Cassani E., Iorio L., Pusani C., Pinelli G., Privitera G., Cesari I., Faierman S.A. (2016). Probiotics and prebiotic fiber for constipation associated with Parkinson disease: An RCT. Neurology.

[114] Georgescu D., Ancusa O.E., Georgescu L.A., Ionita I., Reisz D. (2016). Nonmotor gastrointestinal disorders in older patients with Parkinson’s disease: Is there hope?. Clin. Interv. Aging.

[115] McFarland L.V., Evans C.T., Goldstein E.J.C. (2018). Strain-Specificity and Disease-Specificity of Probiotic Efficacy: A Systematic Review and Meta-Analysis. Front. Med..

[116] Lee J.Y., Kim N., Nam R.H., Sohn S.H., Lee S.M., Choi D., Yoon H., Kim Y.S., Lee H.S., Lee D.H. (2017). Probiotics reduce repeated water avoidance stress-induced colonic microinflammation in Wistar rats in a sex-specific manner. PLoS ONE.

[117] Desbonnet L., Garrett L., Clarke G., Bienenstock J., Dinan T.G. (2008). The probiotic Bifidobacteria infantis: An assessment of potential antidepressant properties in the rat. J. Psychiatr. Res..

[118] Clarke G., Grenham S., Scully P., Fitzgerald P., Moloney R.D., Shanahan F., Dinan T.G., Cryan J.F. (2012). The microbiome-gut-brain axis early in life regulates hippocampal serotonergic system in a sex-dependent manner. Mol. Psy..

[119] Collins F.L., Irwin R., Bierhalter H., Schepper J., Britton R.A., Parameswaran N., McCabe L.R. (2016). Lactobacillus reuteri 6475 Increases Bone Density in Intact Females Only under an Inflammatory Setting. PLoS ONE.

[120] Suez J., Zmora N., Zilberman-Schapira G., Mor U., Dori-Bachash M., Bashiardes S., Zur M., Regev-Lehavi D., Ben-Zeev Brik R., Federici S. (2018). Post-Antibiotic Gut Mucosal Microbiome Reconstitution Is Impaired by Probiotics and Improved by Autologous FMT. Cell.

[121] The Human Microbiome Project Consortium (2012). A framework for human microbiome research. Nature.

[122] The Human Microbiome Project Consortium (2012). Structure, function and diversity of the healthy human microbiome. Nature.

[123] Kalinkovich A., Gabdulina G., Livshits G. (2018). Autoimmunity, inflammation, and dysbiosis mutually govern the transition from the preclinical to the clinical stage of rheumatoid arthritis. Immunol. Res..

[124] Gimblet C., Meisel J.S., Loesche M.A., Cole S.D., Horwinski J., Novais F.O., Misic A.M., Bradley C.W., Beiting D.P., Rankin S.C. (2017). Cutaneous Leishmaniasis Induces a Transmissible Dysbiotic Skin Microbiota that Promotes Skin Inflammation. Cell Host Microbe.

[125] Dinan T.G., Cryan J.F. (2017). Microbes, Immunity, and Behavior: Psychoneuroimmunology Meets the Microbiome. Neuropsychopharmacology.

[126] Shukla S.K., Murali N.S., Brilliant M.H. (2015). Personalized medicine going precise: From genomics to microbiomics. Trends Mol. Med..

[127] Luca F., Kupfer S.S., Knights D., Khoruts A., Blekhman R. (2018). Functional Genomics of Host-Microbiome Interactions in Humans. Trends Genet..

[128] Turpin W., Espin-Garcia O., Xu W., Silverberg M.S., Kevans D., Smith M.I., Guttman D.S., Griffiths A., Panaccione R., Otley A. (2016). Association of host genome with intestinal microbial composition in a large healthy cohort. Nat. Genet..

[129] Bonder M.J., Kurilshikov A., Tigchelaar E.F., Mujagic Z., Imhann F., Vila A.V., Deelen P., Vatanen T., Schirmer M., Smeekens S.P. (2016). The effect of host genetics on the gut microbiome. Nat. Genet..

[130] Wang J., Thingholm L.B., Skiecevičienė J., Rausch P., Kummen M., Hov J.R., Degenhardt F., Heinsen F.A., Rühlemann M.C., Szymczak S. (2016). Genome-wide association analysis identifies variation in vitamin D receptor and other host factors influencing the gut microbiota. Nat. Genet..

[131] Blekhman R., Goodrich J.K., Huang K., Sun Q., Bukowski R., Bell J.T., Spector T.D., Keinan A., Ley R.E., Gevers D. (2015). Host genetic variation impacts microbiome composition across human body sites. Genome Biol..

[132] Khoruts A., Sadowsky M.J. (2016). Understanding the mechanisms of faecal microbiota transplantation. Nat. Rev. Gastroenterol. Hepatol..

[133] Zmora N., Zilberman-Schapira G., Suez J., Mor U., Dori-Bachash M., Bashiardes S., Kotler E., Zur M., Regev-Lehavi D., Brik R.B. (2018). Personalized Gut Mucosal Colonization Resistance to Empiric Probiotics Is Associated with Unique Host and Microbiome Features. Cell.

[134] Zhang H., Sun L. (2018). When human cells meet bacteria: Precision medicine for cancers using the microbiota. Am. J. Cancer Res..

[135] Vrieze A., Van Nood E., Holleman F., Salojärvi J., Kootte R., Bartelsman J.F., Dallinga-Thie G.M., Ackermans M.T., Serlie M.J., Oozeer R. (2012). Transfer of intestinal microbiota from lean donors increases insulin sensitivity in individuals with metabolic syndrome. Gastroenterology.

[136] Kragsnaes M.S., Kjeldsen J., Horn H.C., Munk H.L., Pedersen F.M., Holt H.M., Pedersen J.K., Holm D.K., Glerup H., Andersen V. (2018). Efficacy and safety of faecal microbiota transplantation in patients with psoriatic arthritis: Protocol for a 6-month, double-blind, randomised, placebo-controlled trial. BMJ Open.

[137] Jobin C. (2018). Precision medicine using microbiota. Science.

[138] Egert M., Simmering R., Riedel C.U. (2017). The Association of the Skin Microbiota With Health, Immunity, and Disease. Clin. Pharmacol. Ther..

[139] Perin B. (2018). Autologous skin microbiota transplantation: Development of a technique to transfer representative and viable cutaneous microbial communities. J. Am. Dermatol..

[140] Taverniti V., Guglielmetti S. (2011). The immunomodulatory properties of probiotic microorganisms beyond their viability (ghost probiotics: Proposal of paraprobiotic concept). Genes Nutr..

[141] Kimoto-Nira H., Suzuki C., Kobayashi M., Sasaki K., Kurisaki J., Mizumachi K. (2007). Anti-ageing effect of a lactococcal strain: Analysis using senescence-accelerated mice. Br. J. Nutr..

[142] Tiptiri-Kourpeti A., Spyridopoulou K., Santarmaki V., Aindelis G., Tompoulidou E., Lamprianidou E.E., Saxami G., Ypsilantis P., Lampri E.S., Simopoulos C. (2016). Lactobacillus casei Exerts Anti-Proliferative Effects Accompanied by Apoptotic Cell Death and Up-Regulation of TRAIL in Colon Carcinoma Cells. PLoS ONE.

[143] Konishi H., Fujiya M., Tanaka H., Ueno N., Moriichi K., Sasajima J., Ikuta K., Akutsu H., Tanabe H., Kohgo Y. (2016). Probiotic-derived ferrichrome inhibits colon cancer progression via JNK-mediated apoptosis. Nat. Commun..

[144] Carlson J.L., Erickson J.M., Lloyd B.B., Slavin J.L. (2018). Health Effects and Sources of Prebiotic Dietary Fiber. Curr Dev Nutr.

[145] Markowiak P., Śliżewska K. (2017). Effects of Probiotics, Prebiotics, and Synbiotics on Human Health. Nutrients.

[146] O’Toole P.W., Marchesi J.R., Hill C. (2017). Next-generation probiotics: The spectrum from probiotics to live biotherapeutics. Nat. Microbiol..

